# Therapeutic Perspectives of CD26 Inhibitors in Imune-Mediated Diseases

**DOI:** 10.3390/molecules27144498

**Published:** 2022-07-14

**Authors:** Xiaopeng Hu, Xisheng Wang, Xingkui Xue

**Affiliations:** Medical Research Center, People′s Hospital of Longhua, Shenzhen 518109, China; xiaopenghu661@outlook.com

**Keywords:** CD26/DPP4 inhibitor, immune-mediated diseases, diabetic cardiovascular disease, autoimmune diabetes, inflammatory bowel disease (IBD), graft-versus-host disease (GVHD), coronavirus-related immunological response, multiple sclerosis (MS), anti-tumor immune response

## Abstract

The enzymatic activity of CD26/DPP4 (dipeptidyl peptidase 4/DPP4) is highlighted in multiple studies to play a vital role in glucose metabolism by cleaving and inactivating the incretins glucagon-like peptide-1 (GLP) and gastric inhibitory protein (GIP). A large number of studies demonstrate that CD26 also plays an integral role in the immune system, particularly in T cell activation. CD26 is extensively expressed in immune cells, such as T cells, B cells, NK cells, dendritic cells, and macrophages. The enzymatic activity of CD26 cleaves and regulates numerous chomokines and cytokines. CD26 inhibitors have been widely used for the treatment of diabetes mellitus, while it is still under investigation as a therapy for immune-mediated diseases. In addition, CD26’s involvement in cancer immunology was also described. The review aims to summarize the therapeutic effects of CD26 inhibitors on immune-mediated diseases, as well as the mechanisms that underpin them.

## 1. Introduction

The lymphocyte membrane-bound protein CD26 is the same as DPP4, a serine protease expressed on the luminal and apical cell membranes. CD26 is a 105–110 kDa single-pass type II integral membrane glycoprotein in the form of homodimer. Each monomer displays a cytoplasmic tail at the N-terminus, with only 6 highly conserved amino acids (aa) and 22 aa in the transmembrane region. The extracellular segment is highly N-glycosylated with 738 aa and can be categorized into three regions [1]: (1) The region of N terminal is glycosylated where residues N85 and N219 provide a binding pocket for substrates; (2) The intermediate region is highly enriched in cysteine and enables the interaction with adenosine deaminase (ADA); (3) The c-terminal region (N509–N766) has catalytic activity.

CD26 belongs to the S9B family of serine proteases which also comprises fibroblast activating proteins (FAP): DPP8, DPP9, DPP10, and DPP6. CD26 has been mostly studied among those serine proteases due to its characteristic of ‘moonlight protein’. CD26 is extensively expressed in immune cells, such as CD4^+^ and CD8^+^ T cells, B cells, NK cells, Dendritic Cells, and Macrophages, and is capable of influencing a wide range of cytokines, chemokines, and peptide hormones mediating signal transduction and cascade amplification, as well as performing the enzymatic reaction towards a substrate. It has been found in a variety of organs, such as intestine, liver, pancreas, placenta, and thymus, and the dissolved form of CD26 was detected in plasma and body fluids [2,3].

## 2. CD26 Functions as a Cell Surface Protein and Soluble Enzyme Molecule

Only the homodimeric form of CD26 has enzymatic activity. CD26 participates in many important processes, such as immunomodulation, psycho/neuronal modulation, and physiological activity. CD26 plays a critical role in the development of immune-mediated disorders [3]. CD26 is able to directly activate and stimulate T cells to proliferate in a TCR/CD3-dependent manner through binding with Caveolin-1 [2]. After antigen uptake via caveolae by antigen presenting cells (APCs), caveolin-1 is exposed on the cell surface and aggregates the immunological synaps in lipid rafts. Consequently, caveolin-1 binds to CD26 and is phosphorylated, leading to the dissociation of interleukin IL-1 receptor associated kinase 1 (IRAK-1) and Tollip [4]. NF-κB was then subsequently activated, as well as leading CD86 to be up-regulated, thereby supporting the immunological synapse and T cell co-stimulation [3,4]. CD26 also functions as a receptor for adenosine deaminase (ADA) on lymphocytes [5,6]. CD26 has three functions: ADA binding, peptidase activity, and extracellular matrix binding, all of which can interrupt T-cell proliferation and chemotaxis. The natural substrates of CD26 include several chemokines, thus contributing to the regulation of leucocyte migration. The cleaved proteins have a significant impact on receptor binding, and then induce a downstream cascade amplification reaction [5]. CD26 can separate amino terminal dipeptides from polypeptides containing either L-proline or L-alanine in the penultimate position, removing NH2-terminal dipeptides from proteins. CD26 controls glucose metabolism by rapidly degrading circulating glucagon-like peptide-1 (GLP-1) and glucose dependent insulin otropic peptide (GIP), which are negative for maintaining glucose homeostasis.

**Figure 1:**
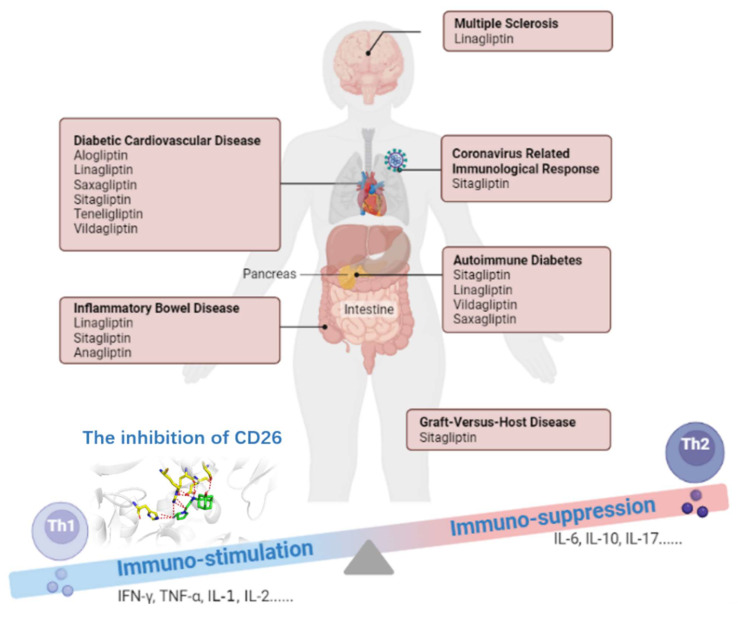
The effect of CD26 inhibitors performed therapeutically in different immune-mediated diseases through a biased Th1 to Th2 cytokine profile. The inhibition of CD26 breaks Th1-cell-mediated immune stimulation, as well as activating the Th2-cell-mediated immune suppression.

**Figure 2:**
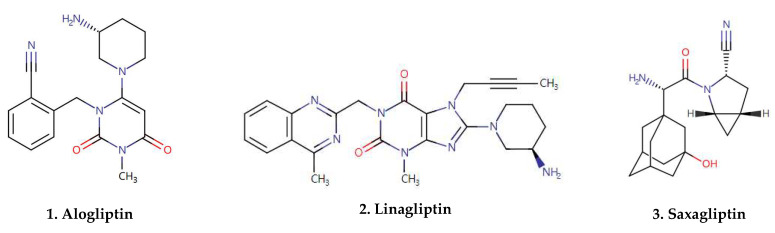

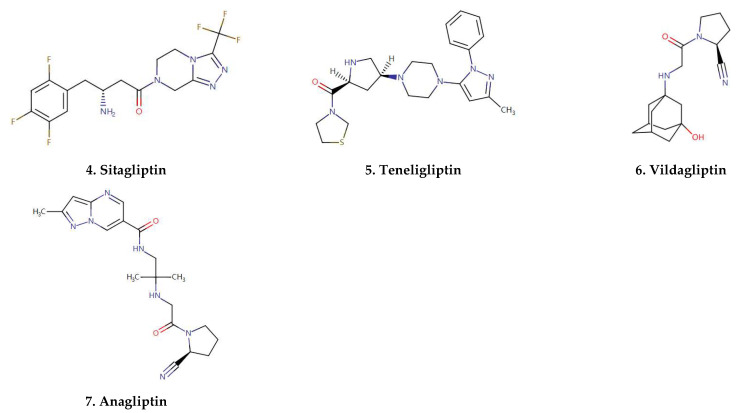
Molecular structures of gliptins.

To summarize, CD26 is characterized as ‘moonlight protein’ with multiple functions as a serine protease, receptor, and costimulatory protein [7]. The alteration of CD26 expression is highly correlated with immune-mediated disorders, such as Diabetic Cardiovascular Disease, Autoimmune Diabetes, Inflammatory Bowel Disease (IBD), acute Graft-versus-Host Disease (GVHD), Coronavirus-related immunological response, Multiple Sclerosis (MS), and Tumor Immune Response. Therefore, CD26 has been identified as a therapeutic target [7] (Figure 1). Currently, there are seven CD26 inhibitors commercially available on the market (e.g., sitagliptin, linagliptin, vildagliptin. and others) [8] (Figure 2). These inhibitors are widely used for the treatment of type 2 diabetes mellitus (T2DM), and their immunological effects have been investigated in immune-mediated disorders (Table 1).

## 3. CD26 Inhibitors in Diabetic Cardiovascular Disease

Diabetic cardiovascular dysfunction is a common diabetes complication. Inflammation linked to the onset and exacerbation of T2DM is a key player in the pathogenesis of diabetic cardiovascular complications. Moreover, the inflammation of mediated myocardial fibrosis, which includes structural heart changes, myocardial cell death, and extracellular matrix protein accumulation, is a hallmark of diabetes-induced myocardial dysfunction [27]. Chronic inflammation plays an important role in the pathogenesis of atherosclerosis. Many of the inflammatory mediators pertinent to atherogenic progression originate from macrophages, monocyte, and endothelial surfaces. CD26 is one kind of inflammatory mediator target in diabetes management, and provides a reduction in the associated cardiovascular risk. The activity of circulating CD26 is correlated with poor cardiovascular outcome and a reduced left ventricular ejection fraction in heart failure patients and animal models [28]. CD26 inhibition may reduce monocyte migration to atherosclerotic plaque in response to TNF-α and soluble CD26, as well as up-regulating the adiponectin expression [29,30]. A recent study demonstrated CD26 inhibition reduces atherosclerosis and inflammation via a reduction in macrophage migration and regulating dendritic cell (DC)/macrophage-mediated adipose tissue inflammation in cardiovascular disease [31]. The inhibition of CD26 was used to increase SDF-1 levels, particularly because SDF-1 is enabled to recruit leukocytes, such as neutrophils, monocytes, T cells, and B cells, as well as other bone marrow derived CXCR4^+^ cells, such as stem cells [32]. The enhanced SDF-1 mediated CXCR-4 activation induced the effective collateral artery growth [33]. The inhibition of CD26 activity and, thus, the stabilization of SDF-1 was suggested to be a promising approach to treat diabetic cardiovascular and peripheral artery diseases 

Various studies have identified the effect of CD26 inhibitors on inflammation relevant to atherosclerosis [34]. Alogliptin inhibits IL-1 and TLR4-mediated IL-6 expression, as well as reducing cholesterol and triglycerides, decelerating atherosclerosis [9,35]. Alogliptin also completed Phase 3 Trials for type 2 diabetes and Mellitus/Acute Coronary Syndrome (ACS) Treatment (NCT00968708). The superiority of alogliptin to a placebo for the primary major adverse cardiac events composite was demonstrated. Linagliptin’s inhibitory effects on xanthine oxidase activity is attributed to its methylxanthine chain which may have clinical potential in cardiovascular disease [36]. The study also demonstrated that the levels of H_2_O_2_ in the myocardium and the number of 8-hydroxyguanosine-positive cells decreased after linagliptin treatment. Notably, linagliptin is an effective strategy to relieve the cardiovascular diseases through suppressing oxidative stress. Saxagliptin reduced CD40 expression in inflammatory monocytes and macrophages implicated in the initiation of atherosclerosis [11]. Sitagliptin is also reported to decrease vascular calcification [37]. Lin et al. reported that sitagliptin also prevents the initiation of arterial calcification by inhibiting the activation of NADPH oxidase and NF-κB, which is followed by a decrease in receptors for advanced glycation end products (RAGE) expression [37]. There is evidence suggesting sitagliptin stimulates the adenosine monophosphate-activated protein kinase (AMPK) pathway and inhibits the mitogen-activated protein kinase (MAPK) pathway, both of which are implicated in inflammation and atherosclerosis [12]. Terasaki et al. found that teneligliptin has anti-atherosclerotic properties via inhibiting foam-cell formation of macrophages in type 1 diabetes. The mechanism is linked with the suppression of CD36 and acyl-coenzyme A: cholesterol acyltransferase-1 (ACAT-1) gene expression, partly by attenuating the harmful effects of the advanced glycation end product (AGEs) [13]. A potential mechanism by which vildagliptin decreases infarct size is a reduction in ROS production, given that mitochondrial dysfunction is caused by the extensive release of H_2_O_2_, as one of the pro-oxidative markers [14]. Moreover, CD26 inhibitors, including sitagliptin and vildagliptin, significantly reduce the levels of LDL cholesterol, triglyceride, and free fatty acid and increase the levels of HDL cholesterol in patients with T2DM, indicating the contribution to the reduction in cardiovascular risk [15].

## 4. CD26 Inhibitors in Autoimmune Diabetes

Autoimmune diabetes is a chronic disorder caused by the autoimmune destruction of insulin-producing β cells in the pancreatic islets [18]. The elevated activity of CD26 in diabetes patients positively correlates with the duration of the disease, while being independent of HbA1c level [38].

Suppression of CD26 also reduced production of IL-2, IL-12, and IFN-γ by T cells and peripheral blood mononuclear cells (PBMC) [38]. Recent research demonstrates that the inhibition of CD26 is able to enhance islet neogenesis, β-cell regeneration, and insulin biosynthesis. The combined sitagliptin–losartan treatment promotes β-cell regeneration via enhanced differentiation of pancreatic progenitor cells. Coincidentally, Mu et al. reported that des-fluoro-sitagliptin (an analog of sitagliptin) increases the number of insulin-positive beta-cells in islets, leading to the normalization of beta-cell mass and the beta-cell-to-alpha-cell ratio [16]. Another CD26 inhibitor, linagliptin, reduces the incidence of diabetes by preventing the autoimmune destruction of pancreatic β-cells in NOD mice [17]. CD26 inhibition deregulates the Th1 immune response, increases secretion of Th2 cytokines, activates Tregs, and prevents IL-17 production. Vildagliptin increases insulin secretion and decreases the extensive peri-insulitis, which was mainly formed by CD3- positive T cells [18]. During a cohort study, saxagliptin was effective in decreasing blood glucose levels in glutamic acid decarboxylase antibody 65 (GADA) positive patients and tended to improve β-cell function during a 24 week follow-up [19]. Zhang et al. also reported that adding 2000 IU/day vitamin D3 to saxagliptin might preserve β-cell function in patients with latent autoimmune diabetes in adults LADA [39].

## 5. CD26 Inhibitors in Inflammatory Bowel Disease (IBD)

Inflammatory bowel diseases (IBDs) are multisystem diseases that occur in many patients, not just in the intestines and gastrointestinal tract, but also in extra intestinal brain tissue [40]. T cells from patients with IBD showed elevated CD26 expression, while the activity of circulating CD26 is decreased [40,41]. As one of the key binding proteins for T-cell activation, CD26 plays a critical role in the pathogenesis of IBD [42,43,44]. Globig et al. demonstrates that enriched Th17 in the human inflammatory lesions expresses high levels of CD26 in IBD patients [45]. Recent studies also suggest that CD26 inhibitor administration is able to reduce the level of colonic inflammation [20,46,47].

Linagliptin lowered the colonic histologic scores and leukocyte invasion through the suppression of secretion of IL-6, TNF-α, and myeloperoxidase and the upregulation of IL-10. [20]. Recently, Ning et al. demonstrates that sitagliptin could attenuate DSS-induced experimental colitis and the inhibition can be attributed to the enhancement of GLP-2 action, and the subsequent protective effects on intestinal barrier by inhibiting epithelial cells apoptosis and promoting their proliferation [21]. In the mouse model of IBD, sitagliptin and anagliptin were shown to attenuate the colitis and facilitate the healing in the lesion sites [21,22].

## 6. CD26 Inhibitors in Acute Graft-versus-Host Disease (GVHD)

Graft-versus-host disease is a severe complication and the main cause of mortality in patients undergoing hematopoietic stem cell transplantation. [48]. The accumulation of CD26^+^ T cells was found in GVHD target organs [49]. Ohnuma et al.’s study demonstrated that human IL-26^+^CD26^+^CD4^+^ T cells are involved in the pathophysiology of pulmonary chronic GVHD. Abrogation of CD26 costimulation by caveolin-1-Ig before or during the early onset of GVHD impeded the development of pulmonary chronic GVHD [50,51]. Administration of humanized anti-human CD26 monoclonal antibodies (mAb) decreased x-GVHD severity and prolonged survival in hu-PBL-NOG mice without the loss of engraftment of human T cells [49,52]. Therefore, the targeting of CD26 in T cells has the potential to be useful in studies of GVHD. CD26^+^ T cells infiltrate the skin and intestinal tract in clinical GVHD, and these results strongly suggest that CD26 assists T-cell migration across the endothelial barrier, as well as a role for CD26^+^ T cells in the pathophysiology of GVHD [52,53]. In patients undergoing hematopoietic stem cell transplantation, gliptins are capable of attenuating a cleavage of Chemokine CXCL12, thus facilitating the homing and engraftment of donor cells.

Sitagliptin completed phase 2 trials for graft-versus-host disease (GVHD)/hematopoietic stem cell transplantation (HSCT) Treatment (NCT02683525). The efficacy of sitagliptin, in combination with tacrolimus and sirolimus for the prevention of acute GVHD after myeloablative allogeneic peripheral-blood stem cell transplantation, was investigated. As a result, CD26 inhibition may reduce the incidence of acute GVHD after allogeneic hematopoietic stem cell transplantation (HSCT) [50]. After myeloablative allogeneic hematopoietic stem cell transplantation, CD26 can be inhibited with sitagliptin to block T-cell activation, resulting in a decreased secretion of pro-inflammatory cytokines [23]. Gliptins are reported to attenuate the cleavage of CXCL12, facilitating the homing and engraftment of donor cells in patients undergoing hematopoietic stem cell transplantation [24]. Farag et al. reported that high-dose sitagliptin enhanced engraftment, compared to historic controls at the same institution, resulting in lower incidences of acute graft-versus-host disease [54].

## 7. CD26 Inhibitors in Coronavirus-Related Immunological Response

Coronavirus disease 2019 (COVID-19) is an infectious disease being spread swiftly. Human CD26 is found to be a coronavirus receptor. The attachment of MERS-CoV to CD26 on the host cell through S protein leads to the appearance of genomic RNA in the cytoplasm. Human-neutralizing antibodies directed against the receptor-binding domain (RBD) of the MERS-CoV Spike protein blocks viral binding to human CD26, thereby inhibiting MERS-CoV infection [55]. The binding of spike protein S1 to CD26 could be blocked with the antibody against MERS-CoV Spike protein, or human ADA and the MERS-CoV infection of cells inhibited through the human CD26 pathway [56]. Moreover, recent findings suggested that SARS-CoV-2 can interact with CD26 in conjunction with ACE2 during the infection procedure [57]. The wide distribution of CD26 in the human respiratory tract facilitates the entrance of the virus into the airway tract and contributes to the development of cytokine storm and immunopathology in the fatal COVID-19 pneumonia [58].

The coronavirus pandemic highlights the importance of understanding shared disease pathophysiology in potentially informing therapeutic choices in individuals with type 2 diabetes (T2D) [57,59]. Sitagliptin is associated with reduced mortality in patients with COVID-19 and with type 2 diabetes when administered at hospital admission [25]. With anti-inflammatory property, the CD26 inhibitors represent a promising and potentially beneficial method for the treatment of CD26.

## 8. CD26 Inhibitors in Multiple Sclerosis (MS)

Multiple sclerosis is a chronic inflammatory demyelinating central nervous system disorder leading to serious neurological deficits [60]. Neuro-inflammation is one of the well-known features in MS patients. Recent study showed that there is an increase in CD26^+^ T cells in the peripheral blood and in the cerebrospinal fluid (CSF) of MS patients with progressive forms of the disease. Kaskow et al. found that the CD26^hi^ memory T cells in MS patients correlates with clinical MS disease severity. [60]. The CD26^hi^ T cell subset manifested an effector-memory phenotype (CD45RO^+^ CCR7^low^) and produced a Th1 and Th17 profile of cytokines under inflammatory conditions. Inhibition of CD26 increased TGF-β1 production, and thus modulates T cell function in the central nervous system [61,62].

The inhibition of CD26 increases immunosuppressive cytokine TGF-β1 secretion, which TGF-β1 has anti-inflammatory effects in situ by suppressing the production of NO, and suppressing TNF-α secretion and autoreactive T cell proliferation in autoimmune encephalomyelitis [63]. In the MS mice model, Linagliptin showed neuroprotective properties against neurodegenerative diseases, and exerted an anti-inflammatory effect in MS by reducing brain TNF-α [64]. A large population cohort study by Seong et al. demonstrated that the risk of incident MS, as well as several other autoimmune diseases, was decreased by CD26 inhibitor, which suggests its potential therapeutic effect in MS [65].

## 9. CD26 Inhibitors in Anti-Tumor Immune Response

CD26 was found as a soluble form in tumor-infiltrating immune cells. The characterization as CD26^neg^, CD26^int^, and CD26^high^ lymphocyte subsets are meaningful towards varying levels of antitumor activity. Bailey et al. reported that CD26^high^ T cells employ key mechanisms, including enhanced stemness and migration, to persist and lyse tumors [66]. Importantly, higher expressions of CD26 are found in a wide variety of tumor entities. It can be expressed on the surface of tumor cells, such as gastrointestinal adenocarcinoma, lung cancer, mesothelioma, and melanoma. Increased CD26 expression leads to an upregulation in the receptor of chemokine CXCL, promoting human astrocytic tumor growth [67].

CD26 also promotes the development of metastases. Ohnuma et al. reported that CD26 expression levels in the tumor were significantly higher in colorectal cancer (CRC) patients bearing distant metastasis than in non-metastatic tumors [68]. The mechanism is ascribing to CD26 binds to collagen and fibronectin, as well as an increasing degradation of the extracellular matrix, such as chemokines and other peptides which are involved in cell regulation, migration and the invasion of metastases, thus facilitating cancer cell invasion and metastasis [69]. Ng et al. also reported that CD26 is an attractive therapeutic target for combating tumor progression to improve the prognosis of CRC patients. The CD26 function in inducing CRC migration, invasion, angiogenesis and metastasis, and the potential involvement of matrix metalloproteinases1 (MMP1) and caveolae (CAV1) in such process were also identified [70].

CD26 degraded CXCL 10 rapidly in the normal physiological state, resulting in decreased recruitment and the migration of CXCR3^+^ T cells into the tumor parenchyma [71]. Therefore, the inhibition of CD26 facilitated post-translational modification of chemokines in T cell-dependent anti-tumor effects. There is robust in vivo data on the anti-tumor activity of anti-CD26 monoclonal antibody in mouse xenograft models [68]. Recently, Nishida et al. also identified CD26 expression on human osteoclasts (OCs), and demonstrated that humanized IgG1 monoclonal antibody targeting CD26, and huCD26 mAb was administrated for the treatment of multiple myeloma [72]. Jang et al. reported that vildagliptin can potentially suppress lung cancer growth through macrophage-mediated NK cell activity [73]. Moreover, the efficacy of naturally occurring and immunotherapy-based tumor immunity can be significantly augmented through a combination of CD26 inhibition and checkpoint blockade therapy [68]. Barreira et al. reported that triple therapy—sitagliptin given in combination with antibodies to both CTLA-4 and PD1—remarkably delayed melanoma tumor growth and improved tumor immunity [71].

## 10. Discussion

The CD26 inhibitor class of medications has established safety [74]. Several completed preclinical studies and clinical trials have evaluated the safety and efficacy of CD26 inhibitors in immune-mediated diseases and anti-tumor immune response [3,65,70]. Neither severe hypoglycemic events nor serious side effects were observed. CD26 inhibitors act as potent immune modulators through the regulation of Th1/Th2 phenotype balance and cytokine production. The immune responses of Th1 cells were braked, IFN-γ, TNF-α, IL-1, IL-2 etc. were suppressed, and Th2 cytokines, such as IL-6, IL-10, and IL-17 etc., were up-regulated after treatment with CD26 inhibitors. Thus, the inhibition of CD26 may slow the progression of immune-mediated diseases by shifting the balance toward anti-inflammatory T cell subsets and cytokines.

A higher expression of CD26 is found in a wide variety of malignancies, which exerts interactions by cleaving other molecules selectively (Incretin hormones, chemokines, and many other peptides). Recent research demonstrated that a tumor promoting or suppressing role can be attributed to CD26, indicating CD26 as a therapeutic target. Interestingly, CD26 has been reported to attenuate anti-cancer immunity via chemokine cleavage, as well as dysregulating macrophage M1/M2 polarization [75]. Therefore, inhibition of CD26 enzymatic activity prevents the chemokine being truncated and increases lymphocyte trafficking into the tumor. However, CD26 in the tumor microenvironment deserves further exploration.

The benefit of CD26 inhibition in immune-mediated diseases and anti-tumor immune response mostly stays in the status of preclinical study. Therefore, prospective, randomized, large-scale clinical trials are needed to deepen the impact of CD26 inhibitors.

## 11. Conclusions

DPP4 (CD26) is widely expressed in immune cells, including T cells, B cells, and dendritic cells, and is involved in the pathogenesis of immune-mediated disorders. With the enzymatic activity, CD26 is able to process a series of chemokines and cytokines by cleaving N-terminal dipeptides. The modulation of other CD26 substrates, especially chemokines, raises the possibility that the use of gliptins with acceptable side effect profiles might be applied beyond the treatment of hyperglycemia. Soluble CD26 is significantly increased and contributes to the pathophysiology in immune-mediated disease, such as diabetic cardiovascular disease, autoimmune diabetes, inflammatory bowel disease, GVHD, coronavirus-related immunological response, multiple sclerosis (MS), and tumor immune response. These findings suggest CD26 is a promising target for the treatment of immune-related disorders and tumors. Experimental studies and clinical trials showed the potential of CD26 inhibitors to prevent or alleviate the development of these diseases related to abnormal immune response. Due to the broad repertoire of CD26 binding protein and substrates, the mechanism for the immunomodulatory actions of CD26 inhibitors in these diseases remain to be clearly elucidated.

## Figures and Tables

**Table 1:** The immunoregulation effects of CD26 inhibitors.

Immune-Mediated Disease	CD26 Inhibitor	Mechanism of the Effects	Ref.
Diabetic Cardiovascular Disease	Alogliptin	Inhibits IL-1 and TLR4-mediated IL-6 expression, as well as reducing cholesterol and triglycerides, decelerating atherosclerosis	[9]
	Linagliptin	Inhibited effects on xanthine oxidase.	[10]
	Saxagliptin	Reduced CD40 expression in inflammatory monocytes and macrophages implicated in the initiation of atherosclerosis	[11]
	Sitagliptin	Stimulates the adenosine monophosphate-activated protein kinase (AMPK) pathway and inhibits the mitogen-activated protein kinase (MAPK) pathway	[12]
	Teneligliptin	Suppression of CD36, acyl-coenzyme A: cholesterol acyltransferase-1 (ACAT-1) gene expression partly by attenuating the harmful effects of advanced glycation end product (AGEs)	[13]
	Vildagliptin	Reduce the levels of LDL cholesterol, triglyceride, and free fatty acid and increase the levels of HDL cholesterol in patients with T2DM; Decreases infarct size is reduction in ROS production, given that mitochondrial dysfunction is caused by the extensive release of H_2_O_2_, as one of pro-oxidative markers	[14,15]
Autoimmune Diabetes	Sitagliptin	Unknown	[16]
	Linagliptin	Inhibits the CD26-mediated stimulation of autoimmune T-cell activation and islet infiltration	[17]
	Vildagliptin	Increase insulin secretion and decrease the extensive peri-insulitis which was mainly formed by CD3-positive T cells	[18]
	Saxagliptin	Unknown	[19]
Inflammatory Bowel Disease	Linagliptin	Inhibits the IL-6/JAK2/STAT3 pathway via downregulating p-JAK2/JAK2 and p-STAT3/STAT3 protein expression and HMGB1/RAGE/NF-κB cascade through lowering HMGB1, RAGE, and p-NF-κB p65/NF-κB	[20]
	Sitagliptin	Enhancement of GLP-2 action and the subsequent protective effects on intestinal barrier by inhibiting epithelial cells apoptosis and promoting their proliferation	[21]
	Anagliptin	Unknown	[22]
Graft-versus-Host Disease	Sitagliptin	Block T-cell activation, resulting in decreased secretion of pro-inflammatory cytokines; attenuate cleavage of CXCL12, facilitating the homing and engraftment of donor cells in patients undergoing hematopoietic stem cell transplantation	[23,24]
Coronavirus-related immunological response	Sitagliptin	Unknown	[25]
Multiple Sclerosis (MS)	Linagliptin	Showed neuroprotective properties against neurodegenerative diseases., exerted an anti-inflammatory effect in MS by reducing brain TNF-α	[26]

## Data Availability

This review collected from open access web-source PubMed.
